# Association of self-reported mother–infant relationship with child and adolescent mental health

**DOI:** 10.1192/bjo.2023.4

**Published:** 2023-02-20

**Authors:** Ida Scheel Rasmussen, Philip Wilson, Gritt Overbeck, Katrine Strandberg-Larsen

**Affiliations:** The Research Unit for General Practice and Section of General Practice, Department of Public Health, University of Copenhagen, Copenhagen, Denmark; The Research Unit for General Practice and Section of General Practice, Department of Public Health, University of Copenhagen, Copenhagen, Denmark; and Centre for Rural Health, Institute of Applied Health Sciences, University of Aberdeen, Aberdeen, UK; Department of Public Health, Section of Epidemiology, University of Copenhagen, Copenhagen, Denmark

**Keywords:** Attention-deficit hyperactivity disorder, autistic spectrum disorder, conduct disorder, epidemiology, personality disorder

## Abstract

**Background:**

The quality of the relationship between mother and infant may have profound implications for the development of a child. Early indicators of psychological vulnerability may allow targeting of support for the child's cognitive, emotional and social development. A challenging mother–infant relationship could be one indicator of risk.

**Aims:**

This study examined variations in psychological well-being and psychopathology among boys and girls according to early maternal perception of the mother–infant relationship.

**Method:**

This study is based on 64 663 mother–infant pairs from the Danish National Birth Cohort, for which data on the mother–infant relationship were collected at 6 months postpartum. Behavioural problems were assessed with the Danish version of the Strengths and Difficulties Questionnaire (SDQ) at child ages 7, 11 and 18 years, and we retrieved information on diagnosed childhood and adolescent psychiatric disorders and prescriptions of psychotropic drugs from Danish registries.

**Results:**

Children in the challenging mother–infant relationship group had higher odds of behavioural problems at age 7 among both boys and girls. The same pattern of elevated estimates was identified for boys across all SDQ domains and for girls in three of five SDQ domains. All associations were attenuated at age 18, but increased odds of behavioural problems still existed. A challenging early mother–infant relationship increased the offspring's risk of being diagnosed with a psychiatric disorder or being prescribed a psychotropic drug before the age of 18.

**Conclusion:**

A challenging self-reported mother–infant relationship was associated with later psychopathological difficulties. Routine clinical enquiry may be useful in identification of future vulnerability.

The relationship between parents and their infants has profound implications for a child's development. A healthy and positive relationship creates an environment that nurtures the infant's physical, cognitive, emotional and social development.^1^ Secure attachment occurs when the child's needs for security, calm and understanding are met and allows for optimal brain development in most circumstances.^2^ Secure attachment is strongly related to numerous positive child outcomes,^3^ whereas insecure attachment often leaves infants less able to handle stress and more vulnerable to other adverse behavioural and emotional outcomes.^4^ Equally importantly, impairments in the infant's ability to self-regulate – evidenced by, for example, prolonged crying, sleep disorders and feeding problems – have been associated with behavioural problems, particularly externalising problems, in childhood and adolescence.^5^ The causal mechanisms and the association between the parent–child relationship and later behavioural problems are neither simple nor direct. Although it is indisputable that the infant's ability to self-regulate affects both the well-being of parents and the parent–infant relationship, it has been demonstrated that parental characteristics including levels of distress and overall psychological functioning are also important factors.^6^ Numerous longitudinal studies have examined the relationship between different dimensions of the parent-child relationship and either behavioural problems or psychiatric disorders in childhood. Results show in general that poor parent–infant attachment and dysfunctional parent–infant relationship are associated with behavioural problems and psychopathology in the offspring.^7,8^ Very few studies have examined the association between the parent–infant relationship and the offspring's behavioural problems and/or psychiatric diagnoses through to adolescence.^9^

## Objective

The primary objective of this study was to explore the association between the maternally perceived mother–infant relationship and the offspring's behavioural problems at different times during childhood and adolescence. The secondary objective was to examine the association between the self-reported mother–infant relationship and the offspring's risk of diagnosed psychopathology before the age of 18. All analyses were carried out separately for boys and girls, as there is a substantial literature describing differences in maternal perceptions of and behaviour towards boys and girls.^10^ There is a significant interaction between gender and maternal behaviour, such that maternal behaviour was more strongly related to attachment security for girls than for boys. At a population level, there are also substantial differences in behavioural measures between boys and girls.^11^

## Method

We used data from the Danish National Birth Cohort (DNBC).^12^ The DNBC is a longitudinal nationwide cohort and includes 96 824 children enrolled during pregnancy, corresponding to around 30% of children born in Denmark between 1996 and 2003. For more information and data available, visit dnbc.dk. The protocol for this study is registered at The Open Science Framework (https://osf.io/pjkuq/).

### Participants

Pregnant women who had their first antenatal visit in general practice between 1996 and 2002 were offered participation in the cohort. After inclusion, the women were interviewed by telephone twice during pregnancy and twice postpartum. The participants were invited to answer follow-up questionnaires when the children were 7, 11 and 18 years of age. All codebooks are available at DNBC.DK. All mother–infant pairs, in which the pregnancy resulted in a live, full-term (gestational age ≥37 completed weeks) singleton birth were eligible for this study (*n* = 88 932). We excluded mother–child pairs without information from 6 months postpartum (*n* = 22 421). In addition, we excluded children with congenital anomalies (*n* = 1078) and those diagnosed with conditions affecting the central nervous system (*n* = 179) or global developmental delay (*n* = 545). We excluded these children because these relatively rare conditions could cause behaviour changes independent of the mother–child interaction. Finally, mothers responding ‘don't know’ or ‘don't want to answer’ to the question regarding their relationship with their child were omitted from the analyses (*n* = 46), leaving a total of 64 663 mother–infant pairs (Supplementary material 1 available at https://doi.org/10.1192/bjo.2023.4).

### Ethics

The authors assert that all procedures contributing to this work comply with the ethical standards of the relevant national and institutional committees on human experimentation and with the Helsinki Declaration of 1975, as revised in 2008. Ethical approval was granted by the Committee on Biomedical Research Ethics under case no. (KF) 01-471/94, and the cohort was approved under ref. no 2008-54-0431 by Danish Data Protection Agency. The pregnant women who participated in the study provided informed written consent for themselves and their children to be followed to their legal age of consent. At 18 years of age, the participants born into the DNBC were contacted about their participation and their rights and given information about withdrawal from the study. This study was approved by the DNBC steering committee (ref. 2020–06) and by the University of Copenhagen with case no. 514-0488/20-3000.

### Measures

#### Mother–infant relationship

Assessment of the maternally perceived mother–infant relationship was based on responses to the question: ‘*How have you experienced the task of taking care of your child*’ at 6 months postpartum. We generated an outcome with two possible values: ‘easy mother–infant relationship’, combining the response options ‘very easy’ and ‘fairly easy’, versus ‘challenging mother–infant relationship’, combining the response options ‘difficult’ and ‘very difficult’ (described in detail elsewhere: https://osf.io/pjkuq/). We chose the terms ‘challenging’ and ‘easy’ in our categorisation, as our measure was self-reported and our question reflects the mother's perception.

#### Behavioural problems: Strengths and Difficulties Questionnaire

Validated Danish versions of the Strengths and Difficulties Questionnaire (SDQ) were completed at 7, 11 and 18 years of age.^13^ The SDQ is a screening tool designed to assess five areas of social–behavioural development in children and adolescents: emotional symptoms, hyperactivity/inattention, conduct problems, peer relationship problems and prosocial behaviour. It can be completed by parents, caregivers, teachers, or children and/or adolescents themselves.^13^ The SDQ comprises 25 questions, each of which is rated on a three-point Likert scale. We analysed behavioural difficulties in the five subscales and the total difficulties scale (summed from the four problem subscales) with two possible values: raised or average. The raised group was defined by cut-off points identifying the 10% with the highest problem score in each follow-up. At age 11, the behavioural problem scores were based on a multi-respondent measure compositing the parent-, teacher- and self-reported responses, whereas the 7 and 18 year follow-ups were based on single-respondent data from a parent and the adolescent, respectively; for more detailed information, see Supplementary material 2.

#### Child psychiatric disorders

We retrieved any records of diagnosed child or adolescent psychiatric disorder from the Danish health registries. All live-born children in Denmark are assigned a unique civil registration number that is linked to the National Patient Register.^14^ We obtained data on psychiatric disorders from both in-patient admissions and out-patient contacts. We were interested first in main diagnoses in any of the following diagnostic groups: any psychiatric disorder (ICD-10: F00-F99); schizophrenia, schizotypal and delusional disorders (ICD-10: F20 – F29); mood disorders, anxiety, dissociative, stress-related, somatoform and other nonpsychotic mental disorders (ICD-10: F30-F45, F48, F93); autism spectrum disorders (ICD-10: F84 not: F84.2-F84.4); personality disorders (F60-F69); hyperkinetic disorders (ICD-10: F90, F98.9); and conduct disorders (ICD-10: F91, F90.1).^15^ Thus, the disorder-specific diagnostic groups were not mutually exclusive. We formed a group of children and adolescents with, presumably, milder mental disorders^16^ who had redeemed two or more prescriptions of psychotropic drugs without being registered with a psychiatric diagnosis (ACT groups: N05, N06A, N06B, N06C).^17^

#### Covariates

Potential confounders were chosen *a priori* based on empirical evidence and on a directed acyclic graph (Supplementary material 3). We included the following variables as potential confounders: parity, maternal psychiatric diagnosis, paternal psychiatric diagnosis, maternal educational level, prenatal alcohol consumption, low weight for gestational age^18^ and colic, in accordance with previous studies.^19^ Information on the potential confounders was obtained by self-report in the interviews or from national registries (described in detail elsewhere: https://osf.io/pjkuq/).

### Statistical analyses

We used logistic regression to estimate the odds of the behavioural problem outcomes in children in each subscale of the SDQ according to the reported mother–infant relationship. All three follow-ups were analysed separately. Selective attrition was adjusted for using inverse probability weighting, where the propensity score was predicted by maternal psychiatric diagnosis, paternal psychiatric diagnosis, parity, marital status, maternal age, alcohol consumption during pregnancy and maternal educational level.

Cox proportional hazards regression was used to estimate hazard ratios for the reported mother–child relationship and subsequent offspring psychiatric diagnoses for each of the diagnostic groups. Person-time follow-up was started at the child's first birthday and ended at first psychiatric diagnosis within each diagnostic group, or alternatively at death, migration or end of follow-up. End of follow-up was the adolescent's 18th birthday or the last date of record linkage, 31 December 2018. Children who immigrated before their first birthday were not included in the analysis. We estimated both crude and adjusted ratios and 95% confidence intervals for all analyses. All analyses were conducted separately for boys and girls.

### Sensitivity and subgroup analyses

We performed sensitivity analyses to examine differences attributable to respondent type at the 11 year follow-up. Information on alcohol consumption in the main analyses was taken in prioritised order from the interview at 12 weeks’ gestation, the 30th week of gestation or postpartum. We conducted an analysis restricted to mothers with information on alcohol consumption reported at 12 weeks of gestation owing to risk of recall bias. We computed estimates separately for primiparous and multiparous mothers with thoughts on potential differences in anxiety or stress levels, leisure, parental confidence, etc.

## Results

Ninety-five per cent of the included mothers reported an easy mother–infant relationship. The 5% of the mothers who reported a challenging mother–infant relationship were younger, less educated, more likely to be nulliparous, more likely to be single or unmarried, and more often had a psychiatric diagnosis compared with mothers reporting an easy mother–infant relationship. Infants in the challenging mother–infant relationships were more likely to be boys, to have had symptoms of colic and to have been born small for gestational age (Table 1).

**Table 1:** Sociodemographic characteristics of study population according to maternally reported mother–infant relationship

	Mother–infant relationship			
	Easy %	Challenging %	Missing *n*	*P*-value^b^
	61 689	2974		
Maternal age, years			–	<0.01
16–19	0.4	0.5		
20–24	8.2	9.2		
25–29	38.7	40.6		
30–34	37.5	37.4		
35–40	14.3	11.8		
40+	0.9	0.6		
Parity			92	<0.01
0	45.7	58.3		
1	37.4	31.2		
≥2	16.9	10.5		
Sex			–	<0.01
Girl	49.6	44.4		
Boy	50.1	55.6		
Maternal psychiatric diagnosis^a^			–	0.01
Not present	96.9	96.1		
Present	3.1	3.9		
Paternal psychiatric diagnosis^a^			–	0.5
Not present	97.4	97.3		
Present	2.6	2.7		
Marital status			–	<0.01
Married	60.4	56.7		
Single/unmarried	38.6	43.3		
Maternal educational status			263	<0.05
Primary school	11.9	13.7		
Vocational	48.0	49.0		
Short cycle	5.3	5.0		
Medium cycle	26.4	25.1		
Postgraduate/ research/PhD	8.5	7.4		
Alcohol consumption during pregnancy			290	0.06
Non-drinking	54.5	56.0		
1–3 units/week	43.1	41.2		
>3 units/week	2.4	2.8		
Child, small for gestational age			853	<0.01
No	95.7	92.6		
Yes	4.3	7.4		
Colic			56	<0.01
No	93.8	62.7		
Yes	6.2	37.28		

a. Psychiatric diagnoses before birth of DNBC child.

b. χ²-test.

We observed a higher prevalence of behavioural problems at 7 and/or at 11 years of age in the group where the mothers reported a challenging mother–infant relationship (Table 2). Behavioural problems at 18 years of age were more evenly divided between the two mother–infant relationship groups. However, the prevalence of at least one psychiatric disorder before the age of 18 or redeemed psychotropic drugs was higher among those with a challenging mother–infant relationship (Table 2).

**Table 2:** Prevalence of increased behavioural problems defined by the total difficulties scale score of the Strengths and Difficulties Questionnaire and child and adolescent psychiatric disorders according to maternally perceived mother–infant relationship

				Mother–infant relationship	
		Cases *N*	Total *N*	Easy %	Challenging %
Behavioural difficulties at 7 years	Boys	1938	20 758	9.02	15.92
	Girls	1999	20 129	9.69	16.01
Behavioural difficulties at 11 years^a^	Boys	1515	15 181	9.66	16.12
	Girls	1626	16 253	9.82	14.26
Behavioural difficulties at 18 years	Boys	1401	13 564	10.22	12.58
	Girls	1832	19 289	9.41	11.48
Psychiatric diagnoses	Boys	3942	32 683	11.86	15.91
	Girls	3919	31 831	12.14	16.41
Psychotropic drugs^b^	Boys	501	32 683	1.47	1.46
	Girls	337	31 831	0.90	1.90

a. Combined measure – *z*-scores.

b. Children/adolescents with redemptions of two or more prescriptions of psychotropic drugs without having a registered psychiatric diagnosis.

### Behavioural difficulties

**Fig. 1:**
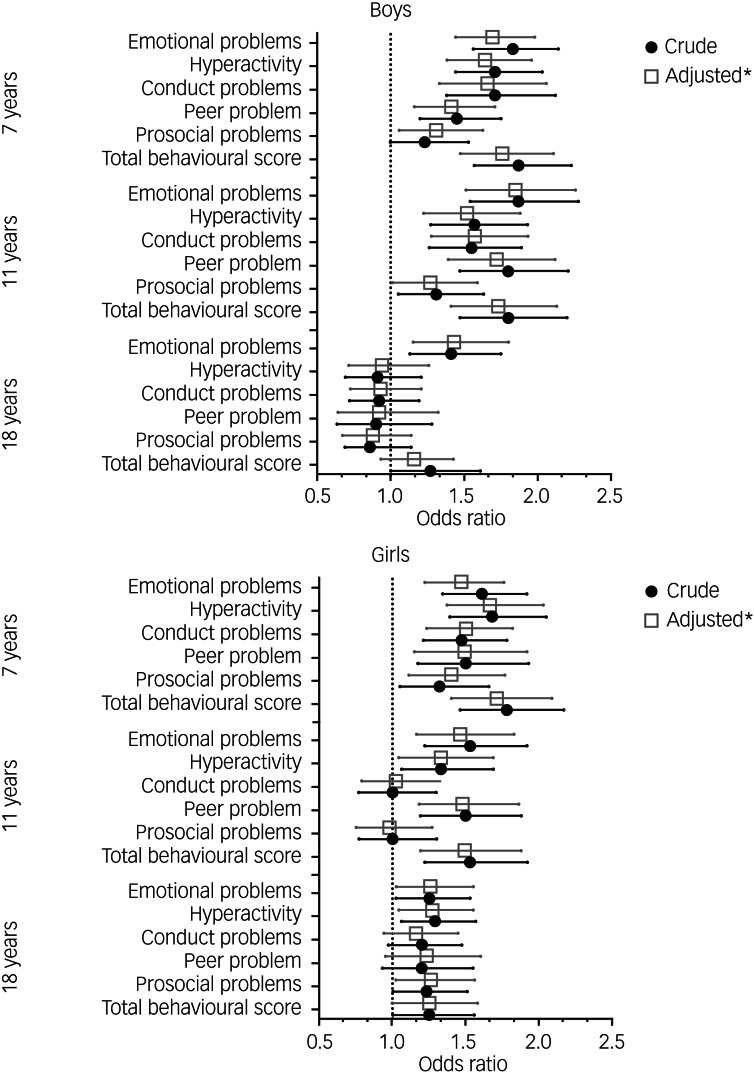
Association between mother–infant relationship at 6 months postpartum and behavioural problems at ages 7, 11 and 18 years, as estimated by logistic regression models. Asterisks indicate values adjusted for parity, maternal psychiatric diagnosis at birth, paternal psychiatric diagnosis at birth, maternal educational level at birth, alcohol consumption, small for gestational age and colic.

A challenging mother–infant relationship was associated with behavioural problems across all five subdomains and for both sexes at age 7 years. Odds of behavioural problems were 1.7-fold higher among both boys and girls when the mother perceived the mother–infant relationship as challenging compared with mothers reporting an easy mother–infant relationship. We found the same pattern of elevated estimates for boys at age 11. However, for girls at 11 years of age we found an elevated risk of emotional problems, hyperactivity and peer problems, but prosocial behaviour and conduct problems were not associated with a challenging mother–infant relation. At 18 years of age, all associations were clearly attenuated, and among boys we only observed indications for increased odds of emotional problems (Fig. 1 and Supplementary material 4). Among girls, all domains had adjusted point estimates above 1 (Fig. 1 and Supplementary material 4). Analyses adjusting for selective attrition based on inverse probability weights (Supplementary material 5) did not change our results notably.

### Psychiatric disorder during childhood and/or adolescence

For both sexes, a challenging early mother–infant relationship was associated with an approximately 30% increased rate of psychiatric diagnoses during childhood and adolescence (Table 3). Among males, the increased rate of any psychiatric disorder was primarily attributable to an increased rate of hyperkinetic disorders, although we found elevated point estimates in schizophrenia, schizotypal and delusional disorders, personality disorders and conduct disorders, all estimated on relatively few cases. Girls in a challenging relationship had increased rates for all diagnostic subgroups of psychiatric disorders compared with those with an easy mother–infant-relationship. The rate was either close to double or greater for hyperkinetic disorders, autism and conduct disorders among the girls, but the latter was based on rare events. For psychiatric problems identified by psychotropic drugs alone, we did not find elevated point estimates for boys in the challenging mother–infant group. We found more than a doubled rate for girls in the challenging mother–infant group.

**Table 3:** Hazard ratios for psychiatric diagnosis according to maternally perceived mother–infant relationship

	Easy mother–infant relationship compared with challenging mother–infant relationship							
	Boys				Girls			
	Cases	HR (95% CI)	HR (95% CI)^a^	Age^b^	Cases	HR (95% CI)	HR (95% CI)^a^	Age^b^
Affective disorders, anxiety and somatoform disorders	1502	1.18 (0.95–1.46)	1.10 (0.88–1.38)	13.48	2681	1.41 (1.19–1.66)*	1.28 (1.08–1.52)*	14.70
Hyperkinetic disorders	1489	1.53 (1.26–1.85)*	1.38 (1.12–1.69)*	11.50	680	1.78 (1.33–2.38)*	1.53 (1.12–2.1)*	13.42
Conduct disorders	319	1.40 (0.91–2.16)	1.18 (0.75–1.85)	10.57	69	2.23 (0.97–5.14)	2.58 (1.08–6.17)	11.53
Autism spectrum disorders	1311	1.13 (0.9–1.43)	1.04 (0.81–1.32)	11.54	695	1.78 (1.33–2.38)*	1.61 (1.19–2.19)*	13.59
Personality disorders	44	2.41 (0.95–6.12)	2.84 (1.06–7.6)*	15.35	202	1.2 (0.64–2.26)	1.18 (0.61–2.27)	15.79
Schizophrenia, schizotypal and delusional disorders	135	1.67 (0.91–3.1)	1.90 (1–3.61)	14.69	277	1.42 (0.86–2.36)	1.31 (0.78–2.21)	15.09
Any psychiatric disorder	3942	1.39 (1.23–1.58)*	1.29 (1.13–1.47)*	14.07	3919	1.39 (1.21–1.59)*	1.28 (1.11–1.48)*	14.07
Psychotropic drugs^c^	481	1.04 (0.69–1.57)	0.88 (0.57–1.36)	11.68	301	2.23 (1.49–3.36)*	2.20 (1.43–3.39)*	13.88

HR, hazard ratio.

a. Adjusted for parity, maternal psychiatric diagnosis at birth, paternal psychiatric diagnosis at birth, maternal educational level at birth, alcohol consumption, small for gestational age and colic.

b. Mean age in years at first diagnosis/initial treatment.

c. Children/adolescents with redemptions of two or more prescriptions of psychotropic drugs without having a registered psychiatric diagnosis.

*P < 0.05.

Adjustments for the selected covariates had a minor impact on the results and most often slightly attenuated the estimated associations.

### Sensitivity and subgroup analyses

When restricting the outcome at age 11 years to parent-reported SDQ scores, we observed a stronger and more significant association between the mother–infant relationship and behavioural problems across all domains except for conduct problems, compared with restricting to either teacher-reported or self-reported SDQ scores for the boys. The results were mixed for the girls; restricting the outcome to self-reported SDQ scores gave a stronger association between the hyperactivity scale and total behavioural problems than restricting the outcome to parent-reported SDQ scores. Likewise, we observed higher odds of emotional and peer problems for parent-reported SDQ scores than for teacher- and self-reported scores (Supplementary material 6). Restricting the analyses to participants with alcohol consumption reported in the earliest pregnancy interview had no impact on our findings. We found no effect of excluding mothers with information on alcohol consumption only reported at 12 weeks of gestation. In the analyses where we examined the children of primiparous and multiparous mothers separately, we observed that the association was marginally stronger for multiparous mothers compared with primipara except for boys aged 7 and girls aged 18 years. We found a stronger association for multiparous mothers compared with primipara in a few of the diagnostic groups, but this finding was not consistent (Supplementary material 7 and 8).

## Discussion

Our findings showed that a challenging mother–infant relationship was associated with increased odds of total behavioural difficulties in childhood at ages 7 and 11 years for both boys and girls in a general population. We found an increased risk of behavioural problems in all subdomains for both sexes at age 7, and for boys at age 11, but only emotional, hyperactivity and peer problems among girls at age 11. The association between both behavioural problems and a challenging mother–infant relationship were attenuated with age and the association became less clear at age 18, although we could not rule out an association for girls. Increased rates of psychiatric diagnoses were observed for the offspring of mothers who had reported a challenging mother–infant relationship. This applied to all of the diagnostic groups for girls but was mainly present for hyperkinetic disorders and conduct problems in boys. A doubling of the receipt of psychotropic drugs was found for girls in the challenging mother–infant relationship group.

The obvious strengths of this study were its prospective design, the large sample size, and the repeated and long-term follow-up data on behavioural problems together with the multi-respondent reporting at 11 years of age. It is also important to highlight that the unique linked administrative data-set allowed us to conduct weighted analyses to compensate for the attrition that exists in all cohorts. Finally, we were able to report both symptoms of behavioural problems and psychiatric diagnoses.

Some limitations are important to consider when interpreting our results. Our biggest concern was our single subjective source of information about the mother–infant relationship causing potential informant bias. This measure is a non-validated source of information about the mother–infant relationship, and it is therefore important to note that this is an overall perception of the relationship between a mother and her infant. We did not consider the background (e.g. feeding problems) of her perception or the actual situation. However, previous studies have provided compelling evidence for an association between mothers’ self-reported relationship with their infant and many aspects of parenting.^20^ The generalisability of the findings is somewhat limited by the underrepresentation of families with low socioeconomic position in the DNBC compared with the Danish population.^21^ We perceived the loss to follow-up as a minor limitation owing to our weighted analyses and previous studies’ conclusions about the low impact of selective drop-out in predictive models of behavioural problems.^22^

This study joins a strong body of research that aims to clarify the link between early mother–infant interaction and/or attachment and the child's risk for psychopathology later in life. In general, our findings are consistent with those of others in suggesting that the mother–infant relationship can be linked to behaviour problems in childhood.^7,8,23^ Other studies have also found an increased risk of receiving psychiatric diagnoses during childhood and adolescence if the mother–infant relationship was challenging.^9^ We have not been able to identify any studies with the same follow-up duration between assessment of mother–infant relationship and the offspring's behavioural problems as that of the present study. We believe that this study is unique as it is the first longitudinal examination indicating some type of long-term association, at least for girls. Previous studies have either focused on behavioural problems during early childhood or have examined diagnosed psychiatric disorders alone.^24–31^

Our findings yield as many questions as answers regarding the maternal role in the development of behavioural problems and psychiatric diagnoses, and there are multiple potential explanations for our findings. Interpretation of our findings is complicated by the mothers’ direct involvement in both the mother–infant relationship assessment at 6 months postpartum and the measurement of behavioural problems at ages 7 and 11 years, which potentially introduced common method bias. Attenuation of the association at 11 years when excluding mother reports, and at age 18 years solely based on self-report, indicate that the prevalence of children's behavioural problems may be overrated among mothers who perceived a challenging mother–infant relationship. We cannot exclude the possibility that the estimates in the main analyses reflect maternal personality to some extent. Gartstein et al (2009) referred to this phenomenon as the depression-distortion effect,^32^ whereas Van der Toorn et al (2010) showed that maternal depression does not bias maternal reporting of children's behavioural problems.^33^ In addition, we could not isolate the extent to which the mother's mental traits, e.g. distress, were internalised by the offspring, thereby influencing the child's self-concept.

The discrepancy between the 7 and 11 year follow-ups in contrast to the 18 year follow-up is likely to be attributable to the lack of direct maternal input to measurement at 18 years of age, but other potential explanations exist. First, our three follow-up assessments covered given periods of life, and the social and educational environment may become more or less well tailored to the individual's needs later in the school system and where cognitive development proceeds apace. Adolescents experiencing behavioural problems may choose circumstances where their behavioural problems are either less important or can be tolerated to a higher degree, which would affect self-reported SDQ scores because comparison is likely to be in relation to peers. Second, adolescents with behavioural problems may join peer groups with similar behavioural difficulties, potentially influencing self-reporting.

Some studies have suggested that emotional regulation problems tend to persist and convert into other regulatory and/or behavioural problems later,^34^ whereas others have discounted this relationship.^35^ With this in mind, we cannot completely reject the possibility that our findings reflect and catch the association between colic, for example, and later psychopathology. Colic was included in our analyses as a potential confounder, and it generally abates before the age of 3 months, so we believe that colic is unlikely to have been the main driver of our findings.

All analyses were conducted separately for girls and boys. Sex-based differences exist in psychopathology^36,37^ and SDQ-scores,^38,39^ and a previous study conducted by Thomson et al demonstrated that male children were more likely to be negatively parented than girls.^40^ Our findings did not reveal large consistent differences between boys and girls when comparing results across the five SDQ domains. However, we found that a challenging mother–infant relationship predicted psychiatric diagnoses in several of the diagnostic groups among girls, whereas a challenging mother–infant relationship mainly predicted hyperactivity among boys. We found slight indications of maternal parity as a moderator, suggesting that multiparous mothers reporting challenging relationships were slightly more likely to have children with later behavioural problems than primiparous mothers.

In summary, our study provides evidence for an association between the early mother–infant relationship as reported by the mother and the child's psychopathology. Despite the limitations, our findings should be considered from a clinical perspective: a dysfunctional self-reported mother–infant relationship, reported as early as infancy, may be an early indicator of potential future social, emotional or behavioural problems, or diagnosed psychopathology. Regardless of the reason for experiencing challenges in early infancy, this may be of importance when targeting resources towards mother–infant dyads in the general population.

## Data Availability

Researchers can apply for use of the data at http://www.dnbc.dk.

## Supplementary material

For supplementary material accompanying this paper visit http://doi.org/10.1192/bjo.2023.4. click here to view supplementary material
