# Supplementary material for: Association of self-reported mother–infant relationship with child and adolescent mental health
Source: BJPsych Open. 2023 Feb 20;9(2):e39. doi: 10.1192/bjo.2023.4 (PMC9970168; doi:10.1192/bjo.2023.4)
Supplement: Supplementary file 1 [file S2056472423000042sup001.doc]

**Supplementary material 1: Flowchart illustrating selection of participants from Danish Birth Cohort**

**
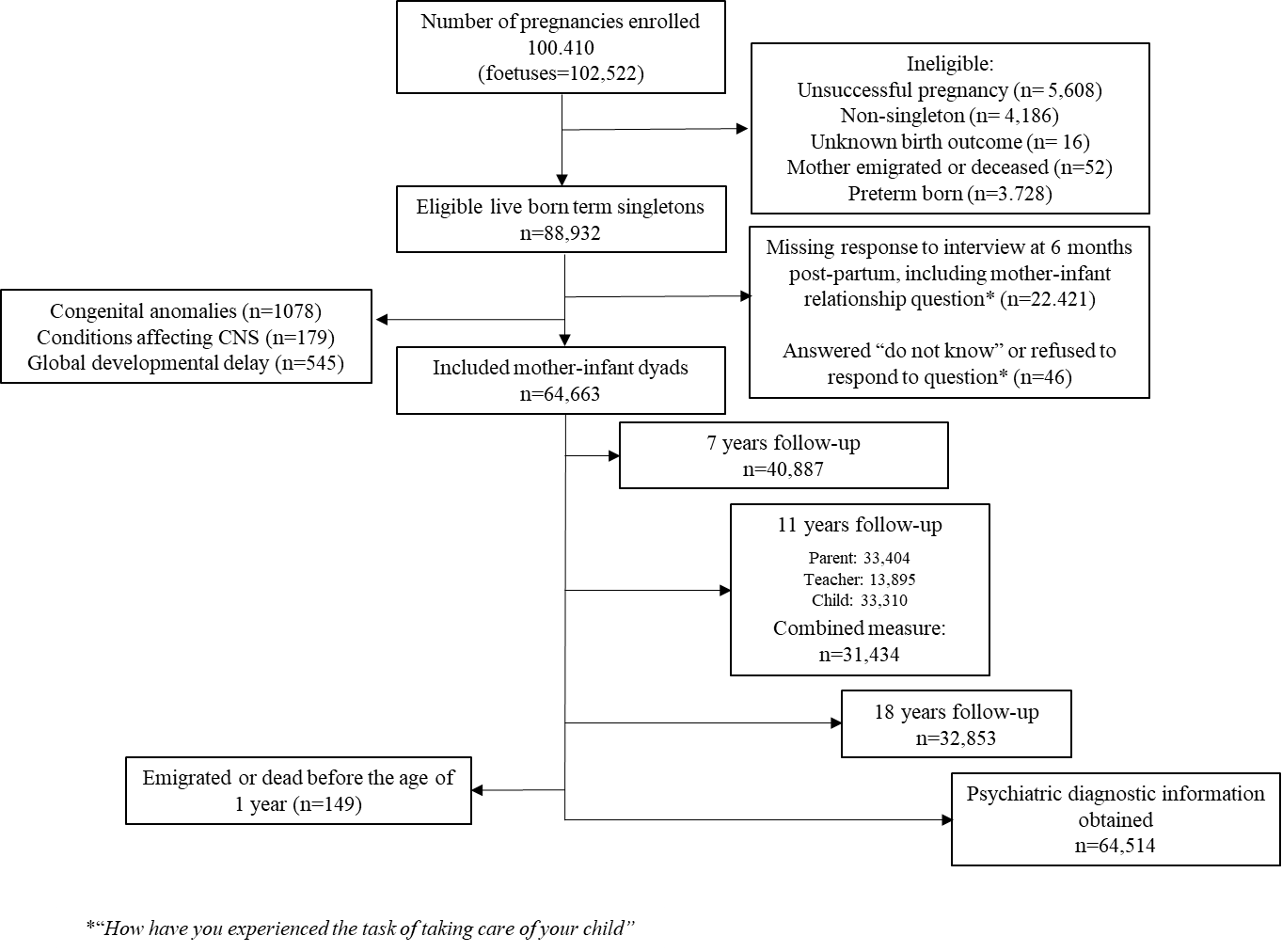
**

**Supplementary material 2: Description of measurement: Behavioural problems, Strengths and Difficulties Questionnaire, 7, 11, and 18 years follow-up**

The Strengths and Difficulties Questionnaire was distributed at 7 years of age to the parents, at 11 years of age to both the parents, the teacher and the child and at 18 years of age to the adolescents. Children can self-report their symptoms from age 11 (1). Multi-informant SDQ scores are desirable for assessing most behavioural problems using the SDQ (2,3).

*7 and 18 years follow-up – single informants reports:*

The SDQ Total Difficulties Scale score (range 0-40) and each subscale score (range 0-10) were coded categorically by cut points. We defined our cut-offs by identifying the 10% with the highest score to generate binary outcomes for raised vs. average. We applied this strategy to both the total behavioral score and the five subscales across all three follow-ups. When the distribution of scores on the five sub-scales did not permit a precise cut-off at the 90th percentile the score closest to the percentile was preferred.

*11 years follow-up – multiple informants' reports*:

To include all accessible data and enhance the validity of the measurement, the data from the three different informants was combined. Domain scores were converted to z scores to standardize and allow comparability across measures. By converting the original scores to z scores, we preserved the distribution of the raw scores (40). Children were categorized as part of the raised problem score-group if their standardized score was above the 90th percentile, calculated for each of the 4 problem scales and the Total Difficulties Scale score, respectively. Similarly, a standardized score below the 10th percentile for the prosocial scale was categorized lowered.

1. SDQ info [Internet]. Available from: https://www.sdqinfo.org/py/sdqinfo/b3.py?language=Danish

2. Madsen KB, Rask CU, Olsen J, Niclasen J, Obel C. Depression-related distortions in maternal reports of child behaviour problems. Eur Child Adolesc Psychiatry. 2020 Mar;29(3):275–85.

3. Johnson S, Hollis C, Marlow N, Simms V, Wolke D. Screening for childhood mental health disorders using the Strengths and Difficulties Questionnaire: the validity of multi-informant reports. Dev Med Child Neurol. 2014 May;56(5):453–9.

**Supplementary material 3: Directed acyclic graph, Model code for http://www.dagitty.net/:**


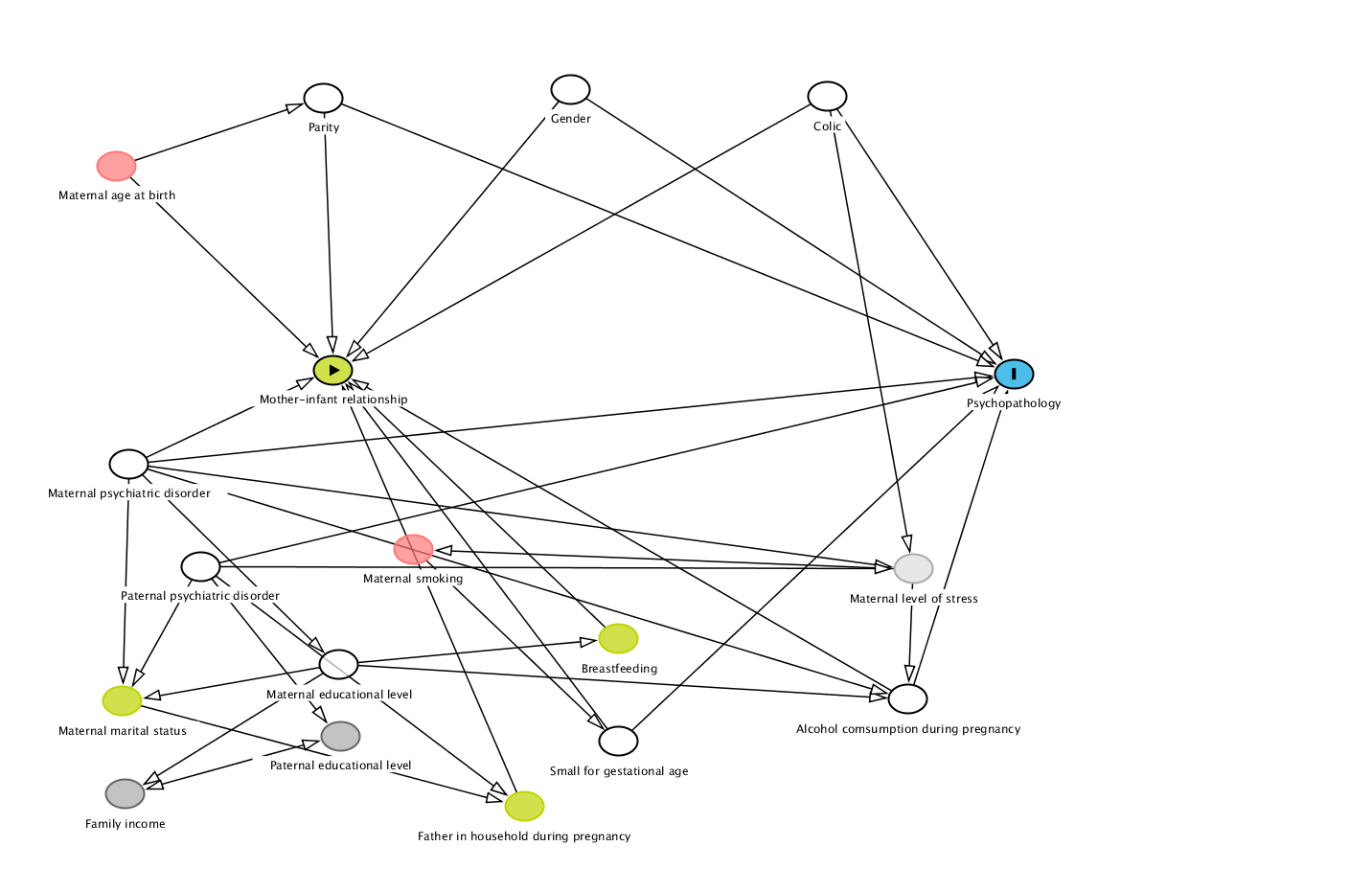


**Supplementary material 4: Association between mother-infant relationship at 6 months post-partum and behavioural problems at age 7, 11 and 18 years of age estimated by logistic regression models.**

|  |  | **Boys** | | | | **Girls** | | | |
| --- | --- | --- | --- | --- | --- | --- | --- | --- | --- |
|  |  | **Crude** | | **Adjusted^a^** | | **Crude** | | **Adjusted^a^** | |
|  | Domain | Odds ratio | 95% CI | Odds ratio | 95% CI | Odds ratio | 95% CI | Odds ratio | 95% CI |
| **7 year** | |  |  |  |  |  |  |  |  |
|  | Emotional problems | 1.83* | 1.56-2.14 | 1.69* | 1.44-1.98 | 1.61* | 1.34-1.92 | 1.47* | 1.22-1.76 |
|  | Hyperactivity | 1.71* | 1.44-2.03 | 1.64* | 1.38-1.96 | 1.68* | 1.39-2.05 | 1.66* | 1.37-2.03 |
|  | Conduct problems | 1.71* | 1.38-2.12 | 1.66* | 1.33-2.06 | 1.47* | 1.21-1.78 | 1.50* | 1.23-1.82 |
|  | Peer problem | 1.45* | 1.2-1.75 | 1.41* | 1.16-1.71 | 1.50* | 1.17-1.93 | 1.49* | 1.15-1.92 |
|  | Prosocial problems | 1.23 | 0.99-1.53 | 1.31* | 1.05-1.63 | 1.32* | 1.05-1.66 | 1.40* | 1.11-1.77 |
|  | Total behavioural score | 1.87* | 1.57-2.23 | 1.76* | 1.47-2.11 | 1.78* | 1.46-2.17 | 1.71* | 1.4-2.09 |
|  |  |  |  |  |  |  |  |  |  |
| **11 year** | |  |  |  |  |  |  |  |  |
|  | Emotional problems | 1.87* | 1.54-2.28 | 1.85* | 1.51-2.26 | 1.53* | 1.22-1.92 | 1.46* | 1.16-1.83 |
|  | Hyperactivity | 1.57* | 1.27-1.93 | 1.52* | 1.23-1.88 | 1.33* | 1.06-1.69 | 1.33* | 1.04-1.69 |
|  | Conduct problems | 1.55* | 1.26-1.89 | 1.57* | 1.28-1.92 | 1.00 | 0.77-1.3 | 1.02 | 0.79-1.33 |
|  | Peer problem | 1.80* | 1.47-2.21 | 1.72* | 1.39-2.12 | 1.50* | 1.19-1.88 | 1.48* | 1.18-1.86 |
|  | Prosocial problems | 1.31* | 1.05-1.63 | 1.27* | 1.01-1.59 | 1.00 | 0.77-1.3 | 0.98 | 0.75-1.27 |
|  | Total behavioural score | 1.80* | 1.47-2.2 | 1.73* | 1.41-2.13 | 1.53* | 1.22-1.92 | 1.49* | 1.19-1.88 |
|  |  |  |  |  |  |  |  |  |  |
| **18 year** | |  |  |  |  |  |  |  |  |
|  | Emotional problems | 1.41* | 1.13-1.75 | 1.43* | 1.15-1.8 | 1.25* | 1.02-1.53 | 1.26* | 1.02-1.55 |
|  | Hyperactivity | 0.91 | 0.69-1.21 | 0.94 | 0.71-1.26 | 1.29* | 1.06-1.57 | 1.27* | 1.04-1.55 |
|  | Conduct problems | 0.92 | 0.72-1.19 | 0.93 | 0.72-1.21 | 1.20 | 0.97-1.47 | 1.16 | 0.94-1.45 |
|  | Peer problem | 0.90 | 0.63-1.28 | 0.92 | 0.64-1.32 | 1.20 | 0.93-1.55 | 1.23 | 0.95-1.60 |
|  | Prosocial problems | 0.86 | 0.69-1.14 | 0.88 | 0.67-1.14 | 1.23 | 1-1.51 | 1.26* | 1.02-1.56 |
|  | Total behavioural score | 1.27 | 1-1.61 | 1.16 | 0.93-1.43 | 1.25* | 1-1.56 | 1.25* | 1-1.58 |

**^a^** Adjusted for parity, maternal psychiatric diagnosis at birth, paternal psychiatric diagnosis at birth, maternal educational level at birth, alcohol consumption, small for gestational age, gender of the child and colic.

* p < 0.05

**Supplementary material 5: Odds ratios of the association between mother-infant relationship at 6 months post-partum and later behavioural problems weighted according to reference population consisting of children of mothers who participated in the 6 months post-partum interview**

|  |  | **Boys** | |  | **Girls** | |
| --- | --- | --- | --- | --- | --- | --- |
|  | **Domain** | **Odds ratio** | **95% CI** |  | **Odds ratio** | **95% CI** |
| 7 year | | | | | | |
|  | Emotional | 1.80* | 1.59-2.04 |  | 1.60* | 1.39-1.84 |
|  | Hyperactivity | 1.70* | 1.49-1.95 |  | 1.68* | 1.44-1.96 |
|  | Conduct problems | 1.68* | 1.42-1.99 |  | 1.47* | 1.26-1.72 |
|  | Peer problem | 1.44* | 1.24-1.68 |  | 1.52* | 1.25-1.85 |
|  | Prosocial problems | 1.25* | 1.05-1.49 |  | 1.34* | 1.11-1.6 |
|  | Total behavioural score | 1.85* | 1.61-2.13 |  | 1.76* | 1.51-2.06 |
|  |  |  |  |  |  |  |
| 11 year | | | | | | |
|  | Emotional | 1.87* | 1.64-2.14 |  | 1.55* | 1.32-1.81 |
|  | Hyperactivity | 1.54* | 1.34-1.78 |  | 1.30* | 1.1-1.53 |
|  | Conduct problems | 1.57* | 1.37-1.8 |  | 0.01 | 0.01-1.19 |
|  | Peer problem | 1.80* | 1.57-2.07 |  | 1.47* | 1.25-1.73 |
|  | Prosocial problems | 1.31* | 1.12-1.52 |  | 0.01 | 0.01-1.19 |
|  | Total behavioural score | 1.80* | 1.57-2.06 |  | 1.51* | 1.28-1.76 |
|  |  |  |  |  |  |  |
| 18 year | | | | | | |
|  | Emotional | 1.39* | 1.21-1.61 |  | 1,24* | 1.06-1.46 |
|  | Hyperactivity | 0.93 | 0.77-1.11 |  | 1,29* | 1.10-1.50 |
|  | Conduct problems | 0.90 | 0.76-1.06 |  | 1,18 | 1-1.39 |
|  | Peer problem | 0.88 | 0.7-1.1 |  | 1,21 | 1-1.47 |
|  | Prosocial problems | 0.87 | 0.74-1.03 |  | 1,23* | 1.04-1.44 |
|  | Total behavioural score | 1.27* | 1.09-1.48 |  | 1,27* | 1.07-1.51 |
|  |  |  |  |  |  |  |

CI, confidence interval.

Inverse probability weighing. Applied variables to predict loss to follow-up included maternal educational level at birth. parity. maternal psychiatric diagnoses. paternal psychiatric diagnoses and maternal age at birth

* p < 0.05

**Supplementary material 6: Association between mother-infant relationship at 6 months post-partum and behavioural problems at 11 years of age restricted to either parent reported SDQ-scores, teacher reported SDQ-scores or self-reported SDQ-scores.**

|  |  | **Boys** | | **Girls** | |
| --- | --- | --- | --- | --- | --- |
|  | **Domain** | **Odds ratio^a^** | **95% CI** | **Odds ratio^a^** | **95% CI** |
| **Parent reported** | |  |  |  |  |
|  | Emotional | 1.80* | 1.47-2.21 | 1.79* | 1.38-2.33 |
|  | Hyperactivity | 2.01* | 1.61-2.5 | 1.38* | 1.09-1.74 |
|  | Conduct problems | 1.30 | 0.94-1.78 | 1.2 | 0.93-1.56 |
|  | Peer problem | 1.51* | 1.26-1.81 | 1.46* | 1.17-1.83 |
|  | Prosocial problems | 1.26 | 0.96-1.65 | 1.18 | 0.96-1.45 |
|  | Total behavioural score | 2.05* | 1.68-2.5 | 1.43* | 1.13-1.8 |
|  |  |  |  |  |  |
| **Teacher reported** | |  |  |  |  |
|  | Emotional | 1.68* | 1.16-2.43 | 1.1 | 0.72-1.67 |
|  | Hyperactivity | 1.53* | 1.1-2.13 | 1.37 | 0.93-2.01 |
|  | Conduct problems | 1.71* | 1.22-2.4 | 0.96 | 0.69-1.33 |
|  | Peer problem | 1.43* | 1.07-1.9 | 1.25 | 0.77-2.03 |
|  | Prosocial problems | 1.13 | 0.69-1.86 | 1.2 | 0.85-1.68 |
|  | Total behavioural score | 1.77* | 1.27-2.48 | 1.14 | 0.77-1.71 |
|  |  |  |  |  |  |
| **Self-reported** | |  |  |  |  |
|  | Emotional | 1.64* | 1.3-2.06 | 1.26 | 0.95-1.67 |
|  | Hyperactivity | 1.34* | 1.04-1.74 | 1.62* | 1.27-2.07 |
|  | Conduct problems | 1.31* | 1.09-1.56 | 1.24 | 0.91-1.69 |
|  | Peer problem | 1.36* | 1.1-1.67 | 1.16 | 0.97-1.4 |
|  | Prosocial problems | 1.04 | 0.82-1.31 | 1.31 | 0.97-1.78 |
|  | Total behavioural score | 1.68* | 1.35-2.09 | 1.52* | 1.19-1.94 |

^a^ Adjusted for parity, maternal psychiatric diagnosis at birth, paternal psychiatric diagnosis at birth, maternal educational level at birth, alcohol consumption, small for gestational age, gender of the child and colic.

* p < 0.05

**Supplementary material 7: Association between mother-infant relationship at 6 months post-partum and behavioural problems at age 7. 11 and 18 years of age restricted to either primiparous or multiparous mothers**

|  |  | **Primiparous** | | | | **Multiparous** | | | |
| --- | --- | --- | --- | --- | --- | --- | --- | --- | --- |
|  |  | **Boys** | | **Girls** | | **Boys** | | **Girls** | |
|  | **Domain** | **Odds ratio^a^** | **95% CI** | **Odds ratio^a^** | **95% CI** | **Odds ratio^a^** | **95% CI** | **Odds ratio^a^** | **95% CI** |
| **7 year** | |  |  |  |  |  |  |  |  |
|  | Emotional | 1.74* | 1.44-2.11 | 1.49* | 1.2-1.84 | 1.56* | 1.17-2.09 | 1.44* | 1.02-2.03 |
|  | Hyperactivity | 1.65* | 1.32-2.06 | 1.48* | 1.15-1.92 | 1.66* | 1.26-2.19 | 2.05* | 1.5-2.79 |
|  | Conduct problems | 1.67* | 1.24-2.24 | 1.52* | 1.18-1.96 | 1.68* | 1.21-2.33 | 1.54* | 1.13-2.08 |
|  | Peer problem | 1.5* | 1.18-1.91 | 1.39* | 1.01-1.93 | 1.26 | 0.91-1.74 | 1.65* | 1.1-2.48 |
|  | Prosocial problems | 1.14 | 0.84-1.55 | 1.36* | 1-1.84 | 1.52* | 1.1-2.09 | 1.47* | 1.02-2.14 |
|  | Total behavioural score | 1.88* | 1.51-2.36 | 1.68* | 1.32-2.15 | 1.56* | 1.14-2.12 | 1.82* | 1.3-2.56 |
|  |  |  |  |  |  |  |  |  |  |
| **11 year** | |  |  |  |  |  |  |  |  |
|  | Emotional | 1.81* | 1.4-2.34 | 1.36* | 1.02-1.82 | 1.87* | 1.36-2.59 | 1.65* | 1.14-2.38 |
|  | Hyperactivity | 1.35* | 1.01-1.8 | 1.28 | 0.93-1.76 | 1.79* | 1.3-2.45 | 1.41 | 0.98-2.03 |
|  | Conduct problems | 1.38* | 1.04-1.83 | 1.02 | 0.72-1.44 | 1.82* | 1.35-2.45 | 1.06 | 0.71-1.58 |
|  | Peer problem | 1.69* | 1.3-2.2 | 1.48* | 1.11-1.98 | 1.76* | 1.25-2.48 | 1.44 | 0.99-2.1 |
|  | Prosocial problems | 1.17 | 0.87-1.58 | 0.98 | 0.7-1.38 | 1.42* | 1.01-2.01 | 0.99 | 0.64-1.53 |
|  | Total behavioural score | 1.64* | 1.26-2.15 | 1.27 | 0.93-1.73 | 1.86* | 1.35-2.56 | 1.85* | 1.31-2.62 |
|  |  |  |  |  |  |  |  |  |  |
| **18 year** | |  |  |  |  |  |  |  |  |
|  | Emotional | 1.31 | 0.97-1.76 | 1.36* | 1.05-1.77 | 1.63* | 1.16-2.29 | 1.11 | 0.79-1.56 |
|  | Hyperactivity | 0.89 | 0.61-1.30 | 1.27 | 0.97-1.65 | 1.07 | 0.68-1.69 | 1.28 | 0.94-1.74 |
|  | Conduct problems | 1.08 | 0.79-1.49 | 1.23 | 0.93-1.62 | 0.74 | 0.48-1.13 | 1.08 | 0.77-1.52 |
|  | Peer problem | 0.86 | 0.53-1.40 | 1.33 | 0.95-1.85 | 1.00 | 0.59-1.71 | 1.09 | 0.72-1.66 |
|  | Prosocial problems | 0.69 | 0.47-1.00 | 1.43 | 1.09-1.88 | 0.69 | 0.47-1.00 | 1.07 | 0.76-1.50 |
|  | Total behavioural score | 1.23 | 0.93-1.62 | 1.37 | 1.02-1.85 | 1.08 | 0.77-1.52 | 1.11 | 0.77-1.60 |

^a^Adjusted for parity, maternal psychiatric diagnosis at birth, paternal psychiatric diagnosis at birth, maternal educational level at birth, alcohol consumption, small for gestational age, gender of the child and colic.
*p < 0.05

**Supplementary material 8: Hazard Ratios for psychiatric diagnosis according to maternally perceived mother-infant relation restricted to either primipara or multiparous**

Table 3. Hazard Ratios for psychiatric diagnosis according to maternally perceived mother-infant relation

|  | **Easy mother-infant relationship compared with challenging**  **mother-infant relationship** | | | | | | | | | | | | |
| --- | --- | --- | --- | --- | --- | --- | --- | --- | --- | --- | --- | --- | --- |
|  | Primiparous | | | | | | | Multiparous | | | | | |
|  | Boys | | | | Girls | | | Boys | | | Girls | | |
|  | Cases | HR^a^ | 95% CI | Cases | | HR^a^ | 95% CI | Cases | HR^a^ | 95% CI | Cases | HR^a^ | 95% CI |
| Affective disorders, anxiety and somatoform disorders | 682 | 1.01 | 0.75-1.38 | 1,243 | | 1.27* | 1.01-1.6 | 771 | 1.21 | 0.87-1.69 | 1,378 | 1.3* | 1-1.68 |
| Hyperkinetic disorders | 668 | 1.27 | 0.95-1.69 | 323 | | 1.48 | 0.97-2.24 | 783 | 1.52* | 1.13-2.04 | 345 | 1.6* | 1-2.57 |
| Conduct disorders | 153 | 0.74 | 0.37-1.49 | 33 | | 0.73 | 0.1-5.38 | 160 | 1.87* | 1.02-3.41 | 35 | 5.43* | 1.95-15.19 |
| Autism spectrum disorders | 643 | 1.02 | 0.74-1.41 | 361 | | 1.51* | 1.02-2.24 | 638 | 1.07 | 0.73-1.56 | 311 | 1.79* | 1.09-2.92 |
| Personality disorders | 14 | 1.72 | 0.23-13.16 | 81 | | 1.36 | 0.54-3.43 | 28 | 3.64* | 1.15-11.48 | 116 | 1.02 | 0.4-2.58 |
| Schizophrenia, schizotypal and delusional disorders | 67 | 1.89 | 0.84-4.27 | 138 | | 1.5 | 0.79-2.83 | 65 | 1.87 | 0.66-5.34 | 133 | 1.02 | 0.41-2.58 |
| Any psychiatric disorder | 1,815 | 1.17 | 0.98-1.4 | 1,813 | | 1.2 | 0.99-1.46 | 2,028 | 1.46* | 1.21-1.77 | 2,019 | 1.38* | 1.12-1.71 |
| Psychotropic drugs^c^ | 225 | 0.74 | 0.4-1.37 | 143 | | 2.5* | 1.47-4.24 | 248 | 1.09 | 0.6-2 | 157 | 1.77 | 0.84-3.73 |

CI, confidence interval. HR, hazard ratio

^a^ Adjusted for parity, maternal psychiatric diagnosis at birth, paternal psychiatric diagnosis at birth, maternal educational level at birth, alcohol consumption, small for gestational age, gender of the child and colic.

^c^ Children/adolescent with redemptions of two or more prescriptions of psychotropic drugs without having a registered psychiatric diagnosis

* p < 0.05

**Supplementary material 9:** Differences in the sociodemographic characteristic of responders and non-responders according to interview at 6 months post-partum including the assessment of mother-infant relationship

|  |  | Mother-infant relationship | |  |  |
| --- | --- | --- | --- | --- | --- |
|  |  | Responders | Non responders | Missing | *p*-value^b^ |
|  |  | % | % | n |  |
|  |  |  |  |  |  |
| Maternal age | |  |  | - | <0.01 |
|  | 16-19 | 1.1 | 0.4 |  |  |
|  | 20-24 | 11.3 | 8.3 |  |  |
|  | 25-29 | 37.6 | 38.7 |  |  |
|  | 30-34 | 36.4 | 37.4 |  |  |
|  | 35-40 | 12.8 | 14.2 |  |  |
|  | 40+ | 0.8 | 0.9 |  |  |
| Parity | |  |  | 317 | <0.01 |
|  | 0 | 48.1 | 46.3 |  |  |
|  | 1 | 36.8 | 37.0 |  |  |
|  | ≥2 | 15.1 | 16.7 |  |  |
| Sex | |  |  |  | <0.01 |
|  | Girl | 48.3 | 49.1 |  |  |
|  | Boy | 51.7 | 50.9 |  |  |
| Maternal psychiatric diagnosis^a^ | | |  |  | <0.01 |
|  | Not present | 96.3 | 96.7 |  |  |
|  | Present | 3.7 | 3.3 |  |  |
| Paternal psychiatric diagnosis^a^ | | |  |  | 0.01 |
|  | Not present | 97 | 97.2 |  |  |
|  | Present | 3 | 2.8 |  |  |
| Marital status | |  |  |  | <0.01 |
|  | Married | 55.1 | 60.1 |  |  |
|  | Single/unmarried | 48.9 | 39.9 |  |  |
| Maternal educational status | |  |  | 480 | <0.01 |
|  | Primary school | 17.0 | 12.2 |  |  |
|  | Vocational | 46.2 | 47.9 |  |  |
|  | Short-cycle | 4.8 | 5.2 |  |  |
|  | Medium cycle | 22.8 | 26.3 |  |  |
|  | Post graduate/ Research/PhD. | 9.2 | 8.4 |  |  |
| Alcohol consumption during pregnancy | | |  | 2215 | <0.01 |
|  | Non-drinking | 55.9 | 54.6 |  |  |
|  | 1-3 units/week | 41.4 | 43 |  |  |
|  | >3 units/week | 2.7 | 2.4 |  |  |

^a^ Psychiatric diagnoses before birth of DNBC child

^b^ Chi-square test
